# The Immunoglobulins of Cold-Blooded Vertebrates

**DOI:** 10.3390/biom4041045

**Published:** 2014-11-24

**Authors:** Rita Pettinello, Helen Dooley

**Affiliations:** School of Biological Sciences, University of Aberdeen, Aberdeen AB24 2TZ, UK; E-Mail: r01rp13@abdn.ac.uk

**Keywords:** immunoglobulin, antibody, ectotherms, cartilaginous fish, bony fish, amphibians, reptiles

## Abstract

Although lymphocyte-like cells secreting somatically-recombining receptors have been identified in the jawless fishes (hagfish and lamprey), the cartilaginous fishes (sharks, skates, rays and chimaera) are the most phylogenetically distant group relative to mammals in which *bona fide* immunoglobulins (Igs) have been found. Studies of the antibodies and humoral immune responses of cartilaginous fishes and other cold-blooded vertebrates (bony fishes, amphibians and reptiles) are not only revealing information about the emergence and roles of the different Ig heavy and light chain isotypes, but also the evolution of specialised adaptive features such as isotype switching, somatic hypermutation and affinity maturation. It is becoming increasingly apparent that while the adaptive immune response in these vertebrate lineages arose a long time ago, it is most definitely not primitive and has evolved to become complex and sophisticated. This review will summarise what is currently known about the immunoglobulins of cold-blooded vertebrates and highlight the differences, and commonalities, between these and more “conventional” mammalian species.

## 1. Introduction

**Figure 1:**
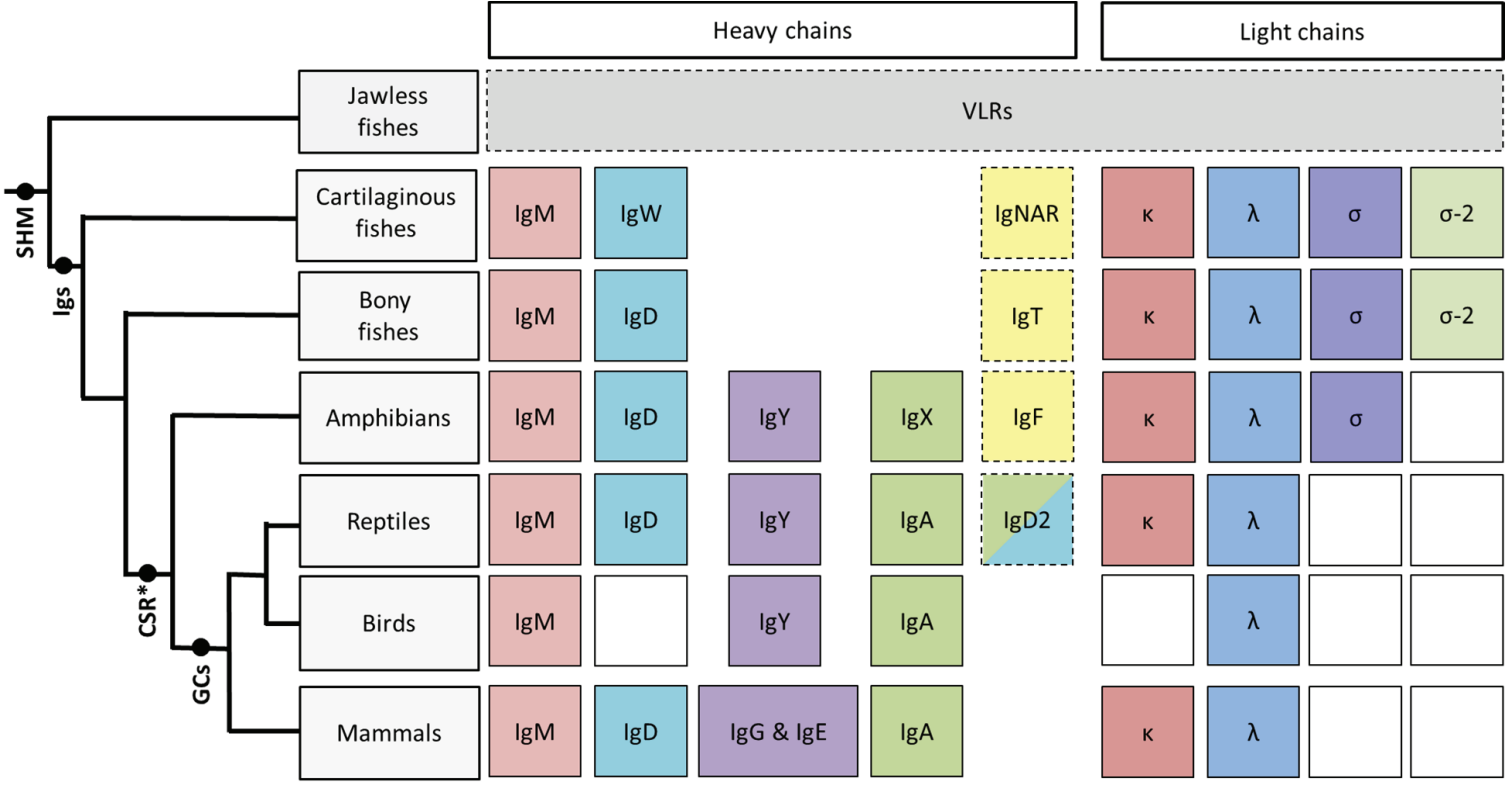
This schematic illustrates the phylogeny of immunoglobulin heavy and light chain isotypes as well as other AIS features as they are currently understood in vertebrates. Except for boxes with broken outlines columns indicate common ancestry; white boxes indicate the isotype has not been found in that particular vertebrate lineage. Although somatic hypermutation (SHM) [14] is present in the jawless fishes, they do not possess Igs, instead they relying upon VLRs for their adaptive response [2]. IgM, IgD, κ and λ isotypes are found in almost every vertebrate lineage. The heavy chain isotype IgY is found from amphibians onwards and is believed to have given rise to both IgG and IgE in mammals [15], while amphibian IgX is both orthologous and functionally analogous to IgA of birds and mammals [16]. Thus far the light chain isotype σ has only been found in cartilaginous fishes, bony fishes and amphibians; σ-2 has recently been identified in representatives of the bony fishes [17] in addition to the cartilaginous fishes after which it was originally named (“σ-cart”) [18]. Conventional class switching (CSR) is first found in amphibians, however recent data from cartilaginous fishes indicates that rearranged V regions from one cluster can be expressed with the constant regions from a different cluster, suggesting an unconventional form of SHM-mediated switch in this lineage [19]. Shark Ig loci lack switch (S) regions and, curiously, switching does not appear to be the unidirectional process that it is in mammals, thus how (or if) this process is directed remains to be clarified. Although primordial germinal centre-like cell aggregates have been observed in bony fishes [20] “classical” germinal centres (GCs) are only found in warm blooded vertebrates (birds and mammals).

While most biologists know that five heavy chain (IgM, IgD, IgG, IgE and IgA) and two light chain (κ and λ) immunoglobulin (Ig) isotypes are found in mammals far fewer are aware that the Igs of the humoral immune system actually have a very long history, reaching back as far as the common ancestor of the jawed vertebrates [1]. Lymphocyte-like cells secreting somatically-recombining receptors (so-called variable lymphocyte receptors or VLRs) have been identified in the jawless fishes (hagfish and lamprey) [2,3], however the mammalian-like adaptive immune system (AIS), based upon somatically-rearranging immunoglobulin superfamily (IgSF) genes is only found in jawed vertebrates. The cartilaginous fishes, which split from the common ancestor with other vertebrates ~450 million years ago (MYA) [4], are the most phylogenetically distant group relative to mammals in which *bona fide* Igs, recombination-activation gene (RAG)-mediated recombination and activation-induced cytidine deaminase (AID)-mediated somatic hypermutation have all been found. Although orthologues of IgM, IgD, κ and λ are found in almost every vertebrate lineage there appears to have been an overall gain of heavy chain isotypes and loss of light chain isotypes as the vertebrate lineage evolved (Figure 1). Recent studies examining the Igs and humoral immune responses of the cold-blooded (ectothermic) vertebrates—cartilaginous fishes, bony fishes, amphibians and reptiles—are not only revealing information about the emergence and roles of the different Ig heavy and light chain isotypes but also the evolution of specialised adaptive features such as isotype switching, somatic hypermutation and affinity maturation. From the data that is accumulating it is becoming increasingly apparent that while the adaptive immune response in these vertebrate lineages may be ancient, it is most definitely not “primitive”.

In this review we will summarise what is known about the Igs and humoral response of cold-blooded vertebrates and try to highlight the differences, and commonalities, between these and their more familiar mammalian counterparts.

## 2. Cartilaginous Fishes

The cartilaginous fishes (Chondrichthyes) diverged from a common ancestor with other vertebrates ~450 million years ago (MYA) [4] and are comprised of two extant subclasses, the Holocephali (chimaeras, such as the elephant fishes, rat fishes and rabbit fishes) and the better known Elasmobranchii (sharks, skates and rays). Thus far three heavy chain isotypes, IgM (μ), IgW (ω; orthologous to IgD in other groups), and the lineage-specific isotype IgNAR [5], as well as four light chain isotypes; kappa (κ), lambda (λ), sigma (σ) and sigma-2 (σ-2; alternatively called σ-cart), have been found in cartilaginous fishes (Figure 1). In mammals B cell maturation occurs in the foetal liver, switching to the bone marrow in the adults; cartilaginous fishes lack bone marrow but instead have two novel organs, the epigonal organ associated with the gonads and the Leydig organ embedded within the walls of the oesophagus, that are the major sites of B cell lymphopoiesis [6]. As in mammals T cell maturation occurs in the cartilaginous fish thymus [6]. Like other ectothermic vertebrates cartilaginous fishes lack lymph nodes [7] and so the adaptive immune response occurs in the spleen and potentially also the gut associated lymphoid tissue (GALT).

**Figure 2:**
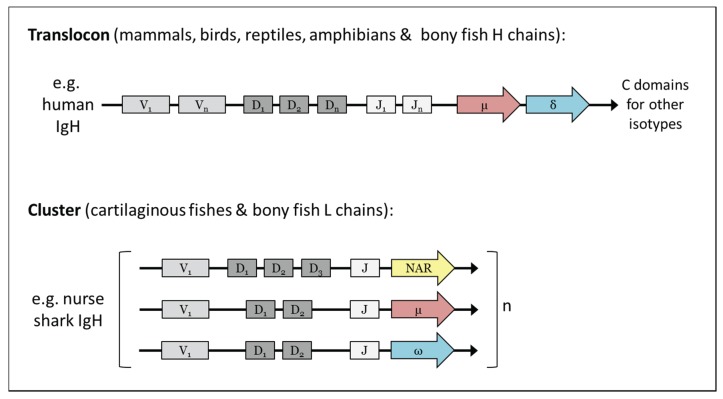
The immunoglobulins of most species are found in the translocon organisation typified by mammals however those of the cartilaginous fishes are found in the so-called “cluster” configuration with only the constant (C) domains for one isotype 3' of a single variable (V) segment, 1–3 diversity (D) segments and a single joining (J) segment [8]; rearrangement occurs almost exclusively within a cluster and not between adjacent clusters [9]. The Igs of bony fishes are mixed, with their heavy chain loci being present in the translocon configuration but the light chain loci in the cluster configuration.

Both the IgH and IgL genes in cartilaginous fishes are arranged in clusters, differing from the translocon organisation typified by mammalian Igs (Figure 2). Each cluster is composed of a variable (V) segment, a number of diversity (D) segments and a joining (J) segment, as well as the constant (C) domains required to generate that particular Ig chain [8]. The exact number of clusters for each isotype varies between species, ranging from a few (2–3) to hundreds, and rearrangement almost exclusively occurs within a cluster rather than between clusters [5,9]. Another characteristic unique to this group is that some Ig clusters are partly (VD-J) or fully (VDJ or VJ) pre-joined in the germline, apparently as a result of RAG expression in germ cells [10,11]; although the antibodies produced by these “germline-joined” clusters have limited CDR3 diversity they appear to have an expression advantage early in development [12,13] and so it has been hypothesised that they play some sort of protective role in neonates and pups.

A protein with characteristics similar to mammalian IgM was first found in shark serum by Marchalonis and Edelman in the 1960’s [21] and it is now known that IgM is one of the most ancient Ig classes, being present in almost every jawed vertebrates thus far examined [22]. In nurse sharks (*Ginglymostoma cirratum*), currently the best studied cartilaginous fish species with respect to humoral immunity, IgM makes up roughly half of the total serum protein and is secreted in equal amounts in two forms, a monomer (mIgM or 7S) and a pentamer (pIgM or 19S) [23]. The two forms of IgM are produced independently, likely by different lineages of B cells [24], with the monomeric form being neither a precursor nor degradation product of the pentameric form [25]. The production of pIgM is dependent upon the expression of J chain [26], however poor conservation of the J chain C-terminal region (integral to secretory component binding in mammals) [27] makes it unlikely that cartilaginous fish IgM is transported across mucosal epithelia in the same manner as mammalian IgM. Typically both the cell-bound and secreted forms of shark IgM contain 4 Cμ domains however another form of IgM (called IgM1_gj_) is the predominant form in neonatal nurse sharks; IgM1_gj_ lacks Cμ2 and so is convergent in structure with mammalian IgG, forms both monomers and dimers in serum and carries a V region that is completely (VDJ) germline-joined [13]. The preferred partner for the IgM1_gj_ H chain is a σ-2 L chain that is also germline-joined, giving this antibody an entirely “pre-determined” binding site [28]. As shark pups age the expression of pIgM and mIgM gradually increase such that they become the dominant forms of IgM present in adult serum [13].

IgW (previously known as IgNARC [29], IgX [30] or IgR [31] in different species of sharks and skates) is the shark orthologue of IgD [22]; two cell bound forms (containing 2 and 4 Cδ) and at least 7 secreted forms (containing 2, 4, 6 or 8 Cδ as well as a 6 Cδ form that lacks a V region (see below)) have been found thus far [32,33,34], all of which are generated by alternate splicing. The role of IgW in sharks is currently unknown however transcript levels are high in the epigonal, pancreas, thymus and gill of multiple species [32,33,34] perhaps suggesting a role in mucosal protection. Additionally, the short (2Cδ) secreted form has a long, cysteine-rich tail that is very different from the secretory tail of other Igs (IgW or any other known isotype) [32] suggesting its effector function is different from the other isoforms. The cluster organisation of shark Ig genes suggests that, unlike other vertebrates, cartilaginous fish probably do not co-express IgM and IgW on the surface of their B cells; indeed, preliminary studies performed on individually sorted peripheral blood lynphocytes (PBLs) from clearnose skate (*Raja eglanteria*) showed most produced transcript for either IgM or IgW. Additionally, for those few that showed dual expression it was not clear if this was due to the presence of doublet cells [35].

In 1995, a third heavy chain isotype was found in cartilaginous fishes and named NAR (for new antigen receptor). IgNAR (not to be confused with IgNARC, now known as IgW) is unusual in many ways, most notably the fact that its heavy chains form homodimers that do not associate with light chains [36]. The membrane bound form of IgNAR has one V domain and either 3 or 5 C domains per heavy chain, whereas the secreted form has 5 C domains, the last four being homologous to those of IgW. The IgNAR V region is also unusual as it is structurally more closely related to IgL or TCR V regions than to other IgH V regions [37] and it appears that IgNAR may have arisen through the invasion of an IgW cluster by the V domain of NAR-TCR (a shark-specific, doubly rearranging T cell receptor) [38]. In the shark species investigated to date there are far fewer (2–20) IgNAR clusters than IgM clusters however the CDR3 of IgNAR varies greatly in length and sequence composition due to the presence of 3 D segments in each cluster, requiring 4 rearrangement events to encode a fully functional V region. Upon exposure to antigen the IgNAR V regions are mutated to an exceptionally high level [39]. The recent publication of the elephant shark (a chimera) genome showed that it encoded approximately 10 IgM genes as well as those for the κ, λ and σ-2 light chain isotypes, however IgW, IgNAR and σ light chain genes were not found [40].

Four light chain isotypes have also been found in cartilaginous fishes, corresponding to κ (previously called NS4 or type III), λ (NS3 or type II), σ and σ-2 (NS5, type I or σ-cart) [18]. Like the heavy chains, the light chain genes are organised in multiple clusters containing a single V, J, and C gene segment. All of the λ genes are germline-joined in the species studied thus far, whereas the other isotypes have a varied mix of split and joined conformations in the different species [5].

Immunisation studies performed in nurse sharks indicate that pIgM provides the “first line” of defence against invading pathogens; due to its large size pIgM is restricted to the intravascular space where it binds its cognate antigen with low-affinity but high-avidity [24,25]. The functional affinity of pIgM binding does not increase significantly over the course of a response. This, and its presence in the serum of neonates [13,41], suggests the production of pIgM is independent of T cell help. In contrast mIgM and IgNAR appear to be the shark functional equivalents of mammalian IgG and, due to their absence in neonatal serum and the long (4–6 month) lag period before an antigen-specific response is observed for these isotypes, it is hypothesised that their production requires T cell help [26,41,42]. Once a primary response had been raised in nurse sharks the antigen-specific IgM and IgNAR titers remained high for prolonged periods (1–3 years) before dropping to pre-bleed levels. If the animal was subsequently boosted with the same antigen without adjuvant then a response was observed in a much shorter time period (4–6 weeks), indicating cartilaginous fish are capable of a memory response [24].

Like other exothermic vertebrates there is evidence that the humoral response is impacted by environmental factors such as temperature, with the nurse shark response being marginally faster in summer than in winter [43]. IgNAR levels are much lower (~0.1–1 mg/mL) than those of IgM in shark serum, likely due to the presence of fewer IgNAR clusters, and it appears to be prone to proteolysis at its flexible hinge regions between the V/C1 and C3/C4 [24,44]. Interestingly in spiny dogfish (*Squalus acanthias*) and small-spotted catshark (*Scyliorhinus canicula*) a multimeric form of IgNAR is also found in serum with the ratio of the two forms varying between species [33,45]; it is not yet known if these IgNAR multimers require J chain for their formation. Thus far the IgW protein has been difficult to identify, likely due to a combination of low serum levels and, as with IgNAR, an apparent sensitivity to proteolysis [46]. *In situ* analysis performed by Castro and colleagues suggests that at least some IgW expressing cells also express J chain [26] however the biological relevance of this finding requires further work.

Although germinal centres have not been found in cartilaginous fishes the white pulp of the spleen has well-defined B and T cell areas [42] strongly suggesting mammalian-like activation of B cells by helper T cells. Following primary repertoire generation cartilaginous fish V regions are somatically mutated to an exceptionally high level (frequently surpassing the upper levels reported for mammalian V regions) in an antigen-driven manner [39]. The replacement-to-silent mutation (R/S) ratios in shark V regions certainly suggest selection of mutant clones [12] but how this relates to affinity maturation of the response requires further investigation. One unusual feature of the mutations found in cartilaginous fish V regions is the mutation of tandem bases; these tandem mutations are not the result of gene conversion (although there is some evidence that gene conversion also occurs in cartilaginous fish V regions) nor consecutive, side-by-side point mutations, but rather a cartilaginous fish-specific, non-templated mutation process [47,48].

The cluster organisation of cartilaginous fish Ig genes and absence of switch regions precludes the same sort of isotype switching as occurs in other vertebrates. However, a series of experiments recently performed by Hsu and colleagues [19,34] showed that in nurse sharks the V region from one cluster can be expressed with the constant regions from a different cluster, despite the fact that some of these are separated by large (>120 kb) distances. Furthermore, unlike mammals, this unconventional form of “switching” is not unidirectional so, for example, IgW V regions have been found associated with IgM constant regions and vice versa. As the process is heightened following immunisation and occurs concurrent with somatic hypermutation it is thought to be mediated by AID, whereby introduced lesions lead to DNA strand breakage and subsequent re-joining [19]. How (or even if) this process is directed remains to be clarified.

## 3. Bony Fishes

The bony fishes (Osteichthyes) are a large, diverse group comprising over 40,000 species that inhabit almost every aquatic environment on earth (fresh water to marine, deep sea to mountain lakes and streams). Osteichthyes appeared ~420 MYA [4] and soon after divided into major two classes; the Sarcopterygii or “lobe-finned” fishes (coelacanths and lungfishes), and the Actinopterygii or “ray-finned” fish, the class to which the vast majority of bony fish belong. The first tetrapods evolved from a Sarcopterygian ancestor in the Devonian period (~390 MYA) [4] thereafter giving rise to the first amphibians. In the bony fish species studied thus far three heavy chain isotypes; IgM, IgD and IgT (τ), and four light chain isotypes; orthologous to κ, λ, σ, and σ-2, have been identified [17,18] (Figure 1). Bony fish heavy chain genes are found predominantly in the translocon configuration typified by mammalian Igs, however their light chain genes are generally arranged in the cluster configuration similar to the Ig genes in cartilaginous fishes [49] (Figure 2). In terms of lymphoid tissues, bony fish have a spleen and thymus but lack lymph nodes and bone marrow. Instead, the anterior region of the kidney (the head kidney) is the main hematopoietic lymphoid tissue, acting as the analogue of the mammalian bone marrow [7].

Bony fish IgM heavy chains, like those of other species, have 4 constant domains and are expressed in both a monomeric, cell surface-bound form and a polymeric secreted form. Although expressed in low amounts in the fish gut and skin IgM is the most prevalent Ig in bony fish serum where it is found as a tetramer, rather than the pentamer found in other vertebrates [50]. Most bony fish lack J chain and so the IgM tetramers are instead held together by a varying number of intra- and intermolecular disulphide bonds; the number of these bonds appears to be governed by the affinity of the B cell receptor (BCR) for its cognate antigen (greater affinity > more disulphide polymerisation) and govern the so-called “redox form” of the secreted tetramer, this in turn is thought to influence the effector function of the molecule [51,52]. Following immunisation bony fish show a substantial increase in IgM titre but only a moderate increase in binding affinity when compared to that of mammals [53,54] and have been shown to elicit a memory response upon second exposure to an antigen [55].

As in mammals, IgM and IgD are co-expressed on the surface of most naïve teleost B cells through the alternative splicing of a single, long RNA transcript. However, unlike mammals, the teleost IgD heavy chains encode a Cμ1 domain between the V and the Cδ domains [56]. As single-positive IgM^+^IgD^−^ cells have been found in some species of fish it is likely that they down-regulate surface IgD expression following antigen selection. Recently a third population of B cells that are IgM^−^IgD^+^ have been found in catfish [57] and trout [58]; this population is reminiscent of a unique subset of IgD single positive human B cells that are found mainly in the upper respiratory mucosa [59]. A large variety of secreted and cell-surface bound IgD isoforms have been found in bony fish [60,61], having wildly different molecular masses in different fish species due to the numerous Cδ domain duplications and deletions that have occurred. For example, Atlantic cod IgD has only 6 C domains (structure: V-Cμ1-(Cδ1-Cδ2)_2_-Cδ7-Tm) but pufferfish has as many as 14 C domains (structure: V-Cμ1-(Cδ1-Cδ2-Cδ3-Cδ4-Cδ5-Cδ6)_2_-Cδ7-Tm) [60]. Although the exact role of secreted IgD is still open to debate experiments conducted simultaneously on human and catfish IgD suggests it is bound with high affinity by an, as yet, unknown receptor on the surface of basophils and that subsequent cross-linking induces the release of antimicrobial, pro-inflammatory and B cell-stimulating factors [62].

**Figure 3:**
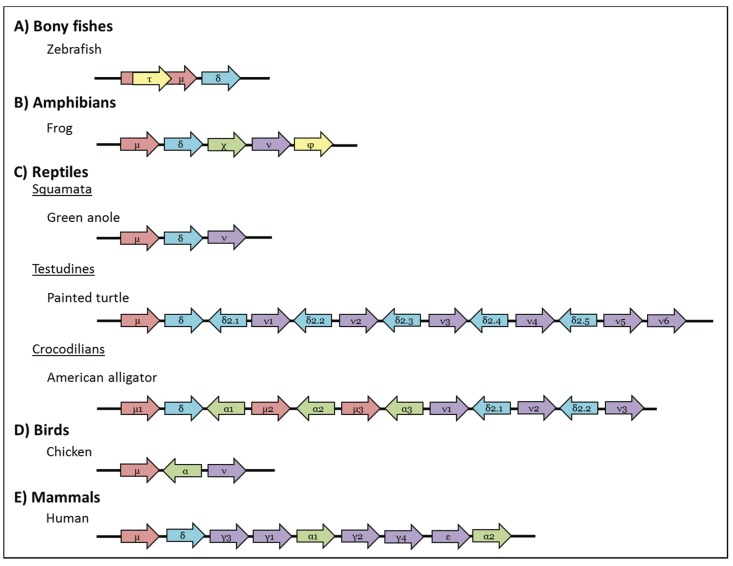
Schematic representation of genomic organisation of the IgH locus in (**A**) Bony fishes: Zebrafish (*Danio rerio*) has a single τ, μ, and δ gene, where the gene encoding IgT is embedded within that of IgM [63,64]; (**B**) Amphibian: The frog (*Xenopus tropicalis*) IgH locus has one gene each for μ, δ, χ, ν, and φ [77]; (**C**) Reptiles: Squamata: The green anole (*Anolis carinolensis*) has only one gene for μ, δ, and ν [78]; Testudines: painted turtles (*Chrysemys picta belli*) have multiple copies of δ2 that are transcribed in the opposite direction to those of μ, δ, ν [79]; Crocodilians: American alligator (*Alligator mississippiensis*) has multiple copies of the μ, α and ν genes and has α- and IgD2-encoding genes that are transcribed in the opposite direction to the other genes [80]; (**D**) Birds: Chickens (*Gallus gallus*) have one copy each of μ, α and ν. As in reptiles the chicken α gene is transcribed in opposite direction to the others [81]; (**E**) Mammals: Humans (*Homo sapiens*) have multiple copies of γ and α; all human IgH genes are transcribed in the same orientation. Arrows indicate the direction of gene transcription and all pseudogenes have been excluded for clarity. Genes and intergenic distances are not drawn to scale.

In 2005, a third heavy chain isotype, IgT (τ; found at the same time in zebrafish and called IgZ) was found in bony fishes [63,64]. IgT shares V segments with IgM but, interestingly, possess its own set of D and J segments that are embedded within the locus for IgM (Figure 3). This means that isotype exclusion for IgM and IgT works through alternative VDJ recombination rather than mRNA splicing (as occurs for the μ and δ chains) or *bona fide* class switch recombination as observed in higher vertebrates [65]. Usually IgT has 4 C domains however some species show slight differences, for example stickleback IgT has three and fugu only two C domains, whilst carp IgT contains a Cμ1 domain similar to bony fish IgD [66]. So far IgT has not been found in medaka or channel catfish so is presumed to be missing from some species [66]. Although present at low levels as a monomer in the serum, IgT is expressed at a much higher levels as a non-covalently associated multimer by B cells present in the gut epithelium; this multimeric form of IgT is transported into the gut lumen by means of the fish polymeric immunoglobulin receptor (pIgR), much like mammalian IgA, and has been found coating the surface of gut-resident bacteria [67]. Consistent with its proposed role in intestinal protection it was found that fish previously infected with the gut parasite *Ceratomyxa shasta* had more IgT^+^ B cells, but the same number of IgM^+^ cells, in their GALT than control (uninfected) fish [67]. More recently IgT has also been implicated in the protection of teleost skin; the skin of bony fishes is a non-keratinized mucosal tissue that is protected by aggregates of immune cells that collectively form the skin-associated lymphoid tissues (SALT). Teleost SALT structurally resembles GALT and is also rich in IgT^+^ B cells that secrete polymeric IgT into the skin mucus where it presumably protects against bacterial invasion [68]. Phylogenetic analysis has failed to resolve the ancestry of IgT [63,64] however it appears to be the most ancient Ig specialised for mucosal protection.

Studies performed in the Indonesian (*Latimeria menadoensis*) and African coelacanth (*Latimeria chalumnae*) indicate the presence of 2 distinct loci for the heavy chain isotype IgW (the coelacanth/cartilaginous fish orthologue of IgD) but so far no loci for either IgM or IgT have been identified in this lineage [17]. As functional studies have never been attempted in coelacanth the effect the loss of IgM has upon immune functioning are completely unknown. In contrast, three IgM loci have been found in the African lungfishes *Protopterus dolloi* and *Protopterus annectens*, in addition to IgW and two novel isotypes, called IgN and IgQ, which appear to be derived from IgW [69,70]. Interestingly, the heavy chain genes in lungfish appear to be organised in a transitional state between the cluster configuration found in the other bony fishes and the translocon configuration found in the tetrapods [70].

As mentioned four types of light chain, that correspond to λ, κ (previously called L1, L3, F or G dependent upon the fish species it was found in), σ (L2) and σ-2 have also been found in bony fishes [18,71]; κ and σ have been found in most fish species investigated but λ, which was originally found in Atlantic cod and channel catfish [72], appears to have been lost in many fish lineages [71]. Recently, the σ-2 isotype, which was thought to be present only in the cartilaginous fishes and so named σ-cart, has also been found in coelacanth [62] suggesting a wider distribution of this isotype than originally thought. The IgL loci in bony fishes form tightly linked clusters and, as in cartilaginous fishes, there are significant differences in the number of loci for each isotype between species [71]. It is notable that, as yet, no pseudo-light chain corresponding to VpreB or λ5 in mammals has been described in bony fish. Thus exactly how ordered Ig chain rearrangement and H chain allelic exclusion is controlled during B cell development in this lineage remains open for debate [66].

Due to their financial worth as food fish the best studied teleost species with regard to their humoral immune response are probably salmon, trout, catfish and cod. The estimated size of their antibody repertoire, organisation of Ig gene segments, the expressed Ig repertoire, IgM serum concentration and serum antibody responses reveal that there are fundamental differences between species [73]. Even though baseline IgM levels vary with both fish size and water temperature for all species examined the salmonids are generally considered “high responders”, producing a reasonably large amount of antigen-specific antibody following challenge. In contrast Atlantic cod generally show a weak antibody response, and then only towards certain antigens [74]; the recent sequencing of the Atlantic cod genome surprisingly showed that this species lacks MHC class II, CD4 and invariant chain (li) [75]. This loss may be partly compensated through an expansion of MHC class I, some of which appear to have gained MHC class II-like functionality [76] allowing cross-presentation of antigens. However it appears this is not quite sufficient to induce a robust humoral response to all antigens that might be encountered.

It has long been thought that antibody diversity in fish is lower than that generated in mammals however recent studies are suggesting that this is not actually the case; indeed recent high-throughput sequencing studies in zebrafish [82], Atlantic salmon [83] and rainbow trout [84] suggest the antibody repertoire in fish is equal to, or may even exceed, that of humans and mice. Castro and colleagues also showed that rainbow trout are able to induce a typical antibody response, with evidence of specific clonal expansions, in their spleen following infection with VHSV (Viral Haemorrhagic Septicaemia Virus), a natural pathogen of this species; IgM showed the greatest repertoire shift post-infection with IgT being involved in the splenic response to a lesser degree and the IgD repertoire being modified only to a very low level [84].

Although it was thought that affinity maturation does not occur in bony fishes immunisation studies conducted with hapten-carrier proteins such as TNP-KLH and FITC-KLH have shown that binding affinities do actually improve somewhat over a 4–6 month period, but to a much lesser degree than in mammals [53,85]. It is assumed that the modest affinity maturation in fish is due to the absence of “mammalian-like” GCs. Despite a recent study that found discrete clusters of AID-expressing cells, interspersed with melano-macrophages, B cells and CD4^+^ T cells, in the spleen and kidney of catfish [20] (highly suggestive of primordial GCs), analysis of fish V region sequences shows no difference in the R/S ratio between the CDRs and framework regions [86]. Thus it appears that although the somatic hypermutation rates are sufficient in bony fish the mutated sequences are not efficiently selected in their “primordial GCs”.

Bony fish AID differs from that of mammals in having a longer cytidine deaminase motif and extensive substitutions in its carboxy-terminal region [87], an area essential for CSR activity in mammals. Despite this bony fish AID is capable of catalysing CSR in AID^−/−^ mammalian B cells [88,89]. Thus AID had the potential to catalyse the class-switching reaction long before the evolution of switch regions and the presence of multiple constant regions in the heavy chain locus.

## 4. Amphibians

The amphibian lineage diverged from the common ancestor with other jawed vertebrates during the Devonian period, about 350 MYA [4]. Today there are three extant amphibian orders; the Caudata (newts and salamanders), Anura (frogs and toads) and Gymnophiona (the limbless caecilians). The clawed frogs of the genus *Xenopus* are currently the best studied model for understanding amphibian immunity; Igs were first found in the serum of frogs and tadpoles over 40 years ago [90,91] however it was only with the sequencing of the *Xenopus* genome that the presence of five heavy chain isotypes; IgM (μ), IgD (δ), IgX (χ), IgY (ν) and IgF (ϕ) [77], and three light chain isotypes; κ, λ and σ [18,92], was finally confirmed (Figure 1). Both the heavy and light chain genes are found in the translocon structure in amphibians [22,77,92] (Figure 2).

IgM is found as a monomer on the surface of *Xenopus* B cells as well as the J chain associated hexamers/pentamers found at high levels serum. The IgM heavy chain has four constant domains and is heavily glycosylated [93].

The gene encoding IgD was found following the release of the *Xenopus tropicalis* genome; its genomic position, immediately 3' to the gene for IgM (Figure 3), along with the absence of an associated promoter or potential switch regions suggests that amphibian IgD is a true homologue of mammalian IgD and is co-transcribed with IgM. Indeed *Xenopus* IgD is mainly found on the surface of mature IgM^+^ B cells [77] with expression being extinguished following B cell activation. Cell surface-bound IgD has eight constant domains and the V domain is spliced directly onto the Cδ1 exon as in all other lineages except for bony fishes [77]. Very low levels of the IgD secreted form are found in serum [94] and there is some evidence from cDNA transcripts that shorter (likely V–Cδ1-Cδ2) IgD forms such as those found in lungfish and various species of cartilaginous fishes may also be present in amphibians [22]. During their recent study of the Chinese giant salamander (*Andrias davidianus*) Zhu and colleagues found a short form of IgD, containing 4 C regions, that had an 18 amino acids, cysteine-containing, “hinge-like” region between the Cδ1 and Cδ2 domains which is thought to confer conformational flexibility, thus facilitating antigen binding and effector function triggering [95].

IgX was first discovered in the serum of *Xenopus* by Hsu and colleagues in 1985, [94]. Later studies indicated that large numbers of IgX-positive B cells were present in the epithelium of the frog gut but were rare in the spleen [96] suggesting IgX may play a role in mucosal immunity. This role was recently confirmed by Du *et al.* who showed that IgX expression was up regulated following oral immunisation [97]. Additionally, experiments on thymectomised frogs showed that expression of IgX is T cell independent, similar to some of the IgA produced in the mammalian intestine [98]. IgX heavy chains have four constant domains, and while sequence similarity originally suggested a resemblance to IgM [96], it now seems reasonably certain that IgX is both orthologous, as well as functionally analogous, to IgA in mammals and birds [16]. Thus is seems that IgX/A lost a constant domain (CH2) and gained a hinge region in the period between the emergence of the bird and mammalian lineages [99]. IgX is found as multimers in frog serum, however, unlike mammalian IgA, it does not associate with J chain [96]. As mammalian IgA requires J chain for its transport across mucosal surfaces by the poly-Ig receptor (pIgR) it is currently unclear how IgX, lacking J chain, is transported into the gut lumen.

The IgY isotype found in amphibians, reptiles and birds is the functional equivalent of mammalian IgG and indeed is thought to be the likely ancestor of both IgG and IgE [15,16]. It is expressed in spleen and liver in a T-dependent manner. In most amphibians IgY has 4 C domains however a number of alternately spliced forms, each lacking one or more C domains, have been recently described in the salamander [95]; one of the splice forms lacks Cν3 and Cν4 and thus has a structure similar to the IgYΔFc form previously described in reptiles and birds (see below). Western blots of salamander serum and previous work on bull frog serum indicate this form is functionally expressed in amphibians [95,100].

During searches of the *X. tropicalis* EST databank a number of “orphan” heavy chain cDNA sequences were found that had a constant region distinct from those of the previously found IgM, IgD, IgX and IgY. Subsequent analysis located an additional heavy chain isotype, IgF, downstream of IgY [77] (Figure 3). The IgF heavy chain has only two constant domains that are homologous to the first and fourth domains of IgY, suggesting that it likely evolved via duplication of the IgY gene (and so may actually be better described as a sub-class of IgY). However, unlike IgY, IgF possess an extra exon that encodes a flexible hinge-like region between the two constant domains [77]. Transcript analysis suggests the presence of both transmembrane and secreted forms of IgF. RT-PCR indicates IgF expression is highest in *Xenopus* spleen, albeit at much lower levels than IgM, IgX and IgY, with very low level expression also in the gut [77].

In addition to *Xenopus* a few studies have been conducted to characterise the Igs and humoral response of Caudata, mainly the Mexican salamander (or axolotl) *Ambystoma mexicanum* and Iberian ribbed newt *Pleurodeles waltl*. To date IgM, IgY and IgX have all been found in the axolotl [101], where IgY seemingly serves as a mucosal Ig for the first ~7 months after which IgX takes over as the major mucosal Ig isotype [101,102]. The Iberian newt expresses at least three heavy chain isotypes, IgM, IgY and IgP (for *Pleurodeles)* [101,103] that has subsequently been identified as the IgD orthologue [95]; like amphibian IgM and IgY, IgP heavy chains have 4 constant domains and transcripts for both membrane-bound and secretory forms have been found. Expression of IgP appears to be limited to the larval newt spleen and decreases significantly post-metamorphosis [103]. Thus far Chinese giant salamander has been shown to express IgM, IgD and IgY and multiple isoforms have been identified for each by transcript analysis and western blotting [95].

Three light chain isotypes, κ (previously called ρ or L1), λ (type III) and σ (L2) have been found in *Xenopus* [18,92]. The light chain genes are located in different genomic regions. The κ gene is composed of multiple clusters of IgJκ segments followed by a single IgCκ gene, in contrast the λ-encoding locus is formed of multiple copies of IgJλ-IgCλ blocks [104].

Amphibians are the oldest vertebrate group to perform bona fide class switching (*i.e.*, where the C regions of an antibody are replaced by those of another isotype by means of an AID-mediated deletional mechanism) [105]; indeed studies conducted in *Xenopus* by Du Pasquier and colleagues in 1997 were instrumental in understanding the mechanism behind antibody class switching [105]. Although the S region sequences required for CSR are divergent between mammals and amphibians (*Xenopus* S regions are AT-rich not G-rich as in mammals), the S regions from *Xenopus* can functionally replace mouse equivalents to mediate class switching *in vivo*. Thus it is probably the secondary structure formed by S regions (single-strand stem-to-loop structures) that enables recognition of these regions for CSR, and not their nucleotide sequence as was previously thought [49,106].

In contrast to mammals the adaptive immune system of *Xenopus* appears very early in development, with RAG transcripts being observed in tadpoles as early as 4 days post-fertilization and pre-B cells being observed in the liver and spleen by day 5. By day 12 antibodies are present in the serum and an antibody-mediated response can be raised although the antibodies are of low affinity and do not mature [107]. Class switching from IgM to IgY does not appear until around day 15 post-fertilisation, likely as a result of improved T cell help [93]. Immunological memory seems to be transferred through metamorphosis from tadpoles and adult frogs, at least in the case of T and B cell memory against DNP-KLH and some graft responses [93]. Conversely, antibodies from immunised adult female frogs can be transported into their eggs, likely offering some protection to the larvae in the period where their adaptive immune system is still developing [108].

As in mammals B cell maturation in amphibians involves AID-mediated somatic hypermutation following antigen stimulation [109]. However, unlike mammals, affinity maturation in amphibians is generally poor; for example, the affinity of IgY antibodies raised against DNP-KLH increased only ~10-fold during a humoral response in *Xenopus*, contrasting to the >10,000-fold increase observed with the same antigen in mammals. Sequence analysis of Ig heavy chains shows that although the rate of somatic mutations is not very different between frogs and mice the mutations in *Xenopus* tended to occur preferentially at guanine-cytosine (GC) base pairs [110] and the R/S ratio in the CDRs was very low. Taken together this suggests that, like bony fishes, it is the selection of mutants that is sub-optimal in *Xenopus*, again due to the simpler organisation of their lymphoid organs and the absence of organised germinal centres [107,109,110]. Despite this an antibody response has been observed in *Xenopus* in response to the chytrid fungus *Batrachochytrium dendrobatidis* (*Bd*), the causative agent of the lethal skin disease chytridiomycosis that has been linked to worldwide amphibian declines; skin mucus from *X. laevis* previously exposed to *Bd* contained significant amounts of pathogen-specific IgX, and to a lesser extent, IgM and IgY. Further, immunisation with heat-killed pathogen induced specific IgM and IgY antibodies in the serum [111]. Whether these antibodies are protective remains to be determined.

Studies that have examined humoral immunity in urodeles suggest their response is unusually poor to a variety of antigens, even when compared with anuran amphibians like *Xenopus*; if antigen-specific antibodies were observed following immunisation the lag period was long (7–12 weeks) and antibody titres were generally low [101]. Subsequent analysis of Ig sequences by Patel and Hsu indicated that axolotl antibodies are of limited heterogeneity, likely a consequence of few N & P additions (despite TdT being present and expressed), a large contribution of the J region to CDR3 and the presence of only a few, very similar, D regions encoded in the germline [112]. As a point of interest the Iberian ribbed newt is the current model organism for studying the effects of spaceflight upon the immune system and is thus far the only animal to have been immunised in space. Through a series of experiments on newts it has been shown that spaceflight has a number of potentially immunosuppressive effects; the newts displayed a significant (3–5 fold) decrease in circulating lymphocyte numbers, altered VH gene usage and reduced levels of somatic hypermutation even after short spaceflights [113].

## 5. Reptiles

The reptilian lineage, composed of birds and non-avian reptiles, diverged from a common ancestor with mammals about 320 MYA [4]. As birds are considered warm-blooded in this review we will focus upon the much lesser characterized Igs of the non-avian reptiles. Reptiles constitute over 7500 species split into four orders; the Crocodilians (crocodiles, alligators, caimans and gavials), Sphenodontia (tuatara), Squamata (lizards and snakes) and Testudines (turtles, tortoises and terrapins) [114]. Antibodies with similar characteristics to mammalian IgM were first found in tuatara (*Sphenodon punctatum*) serum by Marchalonis and colleagues in 1969 [115]. Since then at least four heavy chain classes; IgM (μ), IgD (δ), IgY (ν), and IgA (α) and two light chain classes; κ and λ, have been found in various species of reptile [78,79,116,117]. With the release of the green anole (*Anolis carolinensis*) genome sequence the reptile heavy and light chains were confirmed to be present in the translocon configuration [118]. Reptiles have a thymus, spleen, GALT and bone marrow, but like the previously described cold-blooded vertebrates they appear to lack lymph nodes and germinal centers [7].

The reptilian IgM gene shares the same structure of C domains as other vertebrates and in most cases all four domains are found in transcripts for both secreted and transmembrane forms; the exception is green anole in which a splice form has been found that encodes a cell-bound form lacking the first two C domains (V-Cμ3-Cμ4-Tm) [78,79,117,119]. Although an extra “reptile-specific” cysteine is found at the beginning of Cμ3 in all species examined to date it appears members of the Squamata (anolis, leopard gecko and various snake species) have lost the cysteine in Cμ1 that forms the disulphide bond with light chains [116]; as IgM (and IgY which also lacks a cysteine in Cν1) have been shown to functionally associate with light chains in at least one species of lizard [120], it is presumed that heavy-light chain pairing occurs in a non-covalent manner.

Crocodilians appear to have duplicated parts of the original Ig cluster resulting in three IgM genes (Figure 3); the IgM1 gene is orthologous to mammalian IgM and the IgD gene is found directly 3' of it. In contrast the IgM2 and IgM3 genes are found interspersed with the genes for the other reptile isotypes and are presumed, like them, to be expressed via class switch recombination [80,121]. The three IgM genes seem to have diverged in function since their emergence, with IgM1 forming pentamers/hexamers in crocodilian serum but IgM2 forming tetramers [121]. In the other reptile species examined IgM seems to be present in serum as J chain-associated pentamers [114,122]. Although IgM expression is highest in the blood and spleen [123], J chain transcript is also found in the lung and throughout the intestine suggesting IgM, and likely also IgA, play a role in mucosal immunity in reptiles as in mammals [124].

A single IgD gene has been found 3' of the IgM gene in most reptile species examined, the exception being the leopard gecko which may have two IgD genes [125]. The reptile IgD gene encodes eleven C domains, more like the long forms of IgD found in other cold-blooded vertebrates and not the much shorter IgD molecules of birds and mammals [78,80,123]. Transcript analysis in a number of species shows that a great variety of splice forms are present for IgD; for example, in anole only four Cδ domains are found in the cell bound form [78], however in turtle this form has six Cδ domains [125]. The secretory forms of IgD are even more diverse with transcripts containing 3, 4, 5 and 6 Cδ domains being found in turtle [125] and transcripts with 2, 4 and 7 Cδ domains in alligators and crocodiles [121]. One study has shown that while the crocodilian IgD gene also contains 11 Cδ domains there are in-frame stop codons present in a number of the C domain exons, meaning these are skipped over to give a transcript with 7 C domains [80,121].

A second IgD form, IgD2, which seems to have arisen by partial duplication of IgD and a subsequent recombination with an IgA-like gene, has been found in leopard gecko and, more recently various turtle and crocodile species, but appears to be missing from green anole. IgD2 generally has six C domains (Cδ1-Cδ2-Cδ3-Cδ4-Cα3-Cα4) and is located between IgD and IgY but is translated in the opposite direction, similar to avian IgA [79,80,123]. The IgD2 gene has been duplicated in the painted and soft-shelled turtle so that multiple (5 and 7 respectively) genes are now present; in both species at least two of these are pseudogenes and there have been domain losses and/or internal duplications in some of the others giving genes containing between 4 and 9 C domains [79]. Like the other isotypes IgD2 antibodies are expressed through class switch recombination and their expression profile (high in the liver, cloaca and intestine in leopard gecko [123]), suggests they play a role in mucosal protection as with amphibian and mammalian IgA.

Low molecular weight Igs were detected by Leslie and Clem in reptiles as early as 1972, but it wasn’t until recently that the presence of antibodies similar to chicken IgY were confirmed in turtles, crocodiles and snakes [79,116,117,121,122]. As in amphibians and birds, the ν gene encodes 4 constant domains and is expressed in both membrane-bound and secreted forms [78,79]. Similar to birds and salamander [95,126] secreted IgY is found in two forms in some reptiles, the full-length IgY containing all 4 Cν domains and a truncated IgY(ΔFc) form, lacking the last two domains [78,122]. It is hypothesized that this form is capable of pathogen neutralisation without inducing inflammation, and at least in ducks its levels are elevated towards the end of a humoral response [127]. In all bird and most reptile species examined the two forms are the result of alternative splicing from a single gene, however it has been suggested that some turtle species may have separate genes for the two forms [125]. IgM and IgD are detectable in turtles at 7–10 days post-fertilisation, however IgY is only measurable at ~90 days post-hatching suggesting expression of this isotype is T cell dependent [128].

An IgA-like gene was first identified in leopard gecko and has since been found in crocodiles and alligators, but has been lost in at least some species of turtle, lizard and snake [80,116,121,125,129]. As in birds, reptilian α gene transcription is inverted compared to that of IgM. In leopard gecko and crocodiles IgA is expressed at high levels in the intestine, indicating an evolutionary conserved role in mucosal immunity [121,129].

Two light chain isotypes, λ and κ, have been found in anole lizards [130] however only the λ isotype has been found so far in snakes [116]. The light chain genomic structure is similar to the one described for amphibian and is conserved in almost all tetrapods [104].

Ig switch regions are present in reptiles and they presumably undergo class switch recombination in a similar manner to amphibians and mammals [119,121]. Immunisation studies indicate that the humoral response in reptiles is also relatively slow compared to mammals, with antigen-specific antibodies peaking about 6–9 weeks post-immunisation [122,131,132]. As in bony fishes and frogs, a prolonged IgM response occurs before class switching to another isotype however low-level IgM was still detectable more than 4 months post-immunisation. An antigen-specific IgY response has also been observed in turtles ~6 weeks post-immunisation [131]. Following a second exposure to the same antigen it was possible to detect an anamnestic response in turtles [131] indicating immunological memory. Although somatic modifications have been observed in turtle V regions, to date, there is no clear evidence of affinity maturation in reptile Igs. It is also not currently clear if these modifications are due to somatic hypermutation, the main form of diversification in fishes and amphibians, or gene conversion as is used in birds [119]; further functional studies in reptiles should help resolve these questions.

Reptile Igs have similar functions to those of other vertebrates; agglutination and precipitation have been documented in turtles and snakes [133] and viral neutralisation has been shown in tortoises after exposure with herpesvirus [134]. Complement activation occurs in alligator serum after inoculation with *E. coli* [135]. Yellowed-bellied slider turtle (*Trachemys scripta scripta*) macrophages showed a faster and stronger respiratory burst in the presence of antibody-opsonized *Salmonella* than with untreated bacteria [136]. There is also some evidence that immune cells and tissues in reptiles are influenced by environmental factors, especially ambient temperature, and so the magnitude and timing of their immune response may vary seasonally [137,138].

## 6. Conclusions

Hopefully it is apparent from the above that we have already learnt much from studying the immunoglobulins and humoral responses of the cold-blooded vertebrates (e.g., the studies of CSR in *Xenopus* mentioned above). However, it is also obvious that there is still much work to be done—we have very limited knowledge of humoral immunity in most of these groups, reptiles and amphibians especially, and almost nothing is known of Ig effector functions in any cold-blooded vertebrate. The rapidly increasing number of genomic/transcriptomic sequence datasets from “less conventional” organisms is already accelerating immune gene discovery and with the subsequent development of new reagents and techniques we should finally be able to address some of the currently unanswered questions as regards humoral immunity in cold-blooded vertebrates. The recent discovery of specialised immune organs (thymus and spleen equivalents) and dedicated lymphocyte subsets (B- and T-like cells) in the jawless fishes have forced us to rethink our ideas regarding the emergence of the AIS (recently reviewed by Flajnik, 2014 [139]) but have also shown what a powerful tool such comparative approaches can be to furthering our understanding. Finally, it should be noted that although the immune system of mice mirrors that of human closely, making them the experimental model of choice for many immune studies, there are also major differences between the two species. Importantly for this review the relative frequency of lymphocytes in the blood, the Ig subsets present and B cell signaling pathway components all differ significantly between mice and humans [140]. Thus studies of immune molecules and immune functioning in other vertebrate species, such as those described herein, give us alternative views of the adaptive immune system, allowing us to determine more “general” rules regarding its function and, on occasion, providing a better comparator than mice for some human studies.
